# *Opuntia ficus-indica* cladodes as feedstock for ethanol production by *Kluyveromyces marxianus* and *Saccharomyces cerevisiae*

**DOI:** 10.1007/s11274-014-1745-6

**Published:** 2014-09-24

**Authors:** Olukayode O. Kuloyo, James C. du Preez, Maria del Prado García-Aparicio, Stephanus G. Kilian, Laurinda Steyn, Johann Görgens

**Affiliations:** 1 Department of Microbial, Biochemical and Food Biotechnology, University of the Free State, P.O. Box 339, Bloemfontein, 9300 South Africa; 2 Department of Process Engineering, Stellenbosch University, Private Bag X1, Stellenbosch, 7602 South Africa

**Keywords:** *Opuntia ficus*-*indica*, *Kluyveromyces marxianus*, *Saccharomyces cerevisiae*, Bioethanol, Lignocellulosic biomass, Dissolved oxygen tension

## Abstract

The feasibility of ethanol production using an enzymatic hydrolysate of pretreated cladodes of *Opuntia ficus*-*indica* (prickly pear cactus) as carbohydrate feedstock was investigated, including a comprehensive chemical analysis of the cladode biomass and the effects of limited aeration on the fermentation profiles and sugar utilization. The low xylose and negligible mannose content of the cladode biomass used in this study suggested that the hemicellulose structure of the *O*. *ficus*-*indica* cladode was atypical of hardwood or softwood hemicelluloses. Separate hydrolysis and fermentation and simultaneous saccharification and fermentation procedures using *Kluyveromyces marxianus* and *Saccharomyces cerevisiae* at 40 and 35 °C, respectively, gave similar ethanol yields under non-aerated conditions. In oxygen-limited cultures *K*. *marxianus* exhibited almost double the ethanol productivity compared to non-aerated cultures, although after sugar depletion utilization of the produced ethanol was evident. Ethanol concentrations of up to 19.5 and 20.6 g l^−1^ were obtained with *K*. *marxianus* and *S*. *cerevisiae*, respectively, representing 66 and 70 % of the theoretical yield on total sugars in the hydrolysate. Because of the low xylan content of the cladode biomass, a yeast capable of xylose fermentation might not be a prerequisite for ethanol production. *K*. *marxianus*, therefore, has potential as an alternative to *S*. *cerevisiae* for bioethanol production. However, the relatively low concentration of fermentable sugars in the *O*. *ficus*-*indica* cladode hydrolysate presents a technical constraint for commercial exploitation.

## Introduction

*Opuntia ficus*-*indica,* commonly known as the prickly pear cactus, is a drought-resistant plant commonly found worldwide in arid and semi-arid regions. Aided by several adaptation mechanisms, including nocturnal stomatal opening and a special CO_2_ fixation pathway known as crassulacean acid metabolism, *O*. *ficus*-*indica* can tolerate a wide range of edaphic and climatic conditions (Russell and Felker 1987). *O*. *ficus*-*indica* possesses up to a fivefold greater efficiency of water utilization than C_4_ plants such as corn and sugar cane, giving it the possibility to produce up to 50 tonne dry mass ha^−1^ year^−1^ and its cultivation requires a low agronomic input (Inglese et al. 2002; Nobel 2002). With increasing focus on the utilization of inexpensive lignocellulosic biomass for the production of bioethanol, the cladodes (the “leaves”, which in fact are the stems) of *O*. *ficus*-*indica* might serve as a second generation feedstock for the production of bioethanol, without competing for prime agricultural land or significantly replacing natural vegetation.

The conversion of lignocellulosic biomass to ethanol usually requires some form of pretreatment prior to enzymatic hydrolysis and fermentation (Hahn-Hägerdal et al. 2006). Enzymatic hydrolysis and fermentation of pretreated feedstocks can either be performed in sequence, i.e. separate hydrolysis and fermentation (SHF), or in one step as a simultaneous saccharification and fermentation (SSF) process. SSF decreases operating costs, since both hydrolysis and fermentation are performed in a single reactor. Moreover, end-product inhibition of the hydrolytic enzymes is minimized due to the removal of inhibitory sugars through fermentation occurring concurrently with enzymatic hydrolysis, thereby allowing a higher solids loading (García-Aparicio et al. 2011; Olofsson et al. 2008). While *Saccharomyces cerevisiae* remains the most established micro-organism used in existing large-scale ethanol industries, the optimum fermentation temperature range (25–35 °C) of this yeast is sub-optimal for SSF due to the higher temperatures required for optimal enzymatic hydrolysis (Bollók et al. 2000; Sun and Cheng 2002). Fermentation at elevated temperatures is also desirable because of the lower cooling costs while minimizing the risk of microbial contamination and also facilitating continuous ethanol recovery (Abdel-Banat et al. 2010; Fonseca et al. 2008). Strains of the thermotolerant yeast *Kluyveromyces* *marxianus* can produce ethanol at temperatures above 40 °C and some have a maximum growth temperature of up to 52 °C (Banat and Marchant 1995; Nonklang et al. 2008). Furthermore, *K*. *marxianus* can utilize a greater range of lignocellulosic sugars than natural strains of *S*. *cerevisiae*, such as cellobiose, xylose and arabinose, and also has a higher growth rate (Lane and Morrissey 2010). *K*. *marxianus* is, however, less ethanol tolerant than *S*. *cerevisiae* and requires oxygen to utilize some sugars (Ginestra et al. 2009). Unlike *S*. *cerevisiae, K*. *marxianus* is Crabtree-negative, cannot grow under strictly anaerobic conditions and ethanol production is almost exclusively linked to oxygen-limited conditions (Bellaver et al. 2004; Fonseca et al. 2008). Nevertheless, its advantages of high growth rate, high temperature tolerance and wide range of sugar utilization render *K*. *marxianus* a potential alternative to *S*. *cerevisiae* for the ethanolic fermentation of lignocellulosic hydrolysates.

In this study a *K*. *marxianus* isolate and a strain of *S*. *cerevisiae*, which served as benchmark, were used to determine the feasibility of utilizing *O*. *ficus*-*indica* cladodes as feedstock for the production of bioethanol. Both SHF and SSF process configurations for the fermentation of enzymatic hydrolysates of cladodes pretreated with dilute acid were evaluated at 40 °C using *K*. *marxianus*. These fermentations were carried out under non-aerated as well as under oxygen-limited conditions. The fermentation profiles of *K*. *marxianus* and *S*. *cerevisiae* were also determined in a chemically defined medium containing sugars similar in concentration to those in an enzymatic hydrolysate of *O*. *ficus*-*indica* cladodes.

## Materials and methods

### Raw material and compositional analysis

Fresh cladodes of the “Algerian” cultivar of *O*. *ficus*-*indica* were harvested from a prickly pear cactus plantation outside Bloemfontein, South Africa. The cladodes were cut into strips using a mechanical shredder and sun-dried, after which they were further processed by hammer milling to a particle size of 1 mm. The dried and milled cladode biomass was thoroughly mixed to ensure representative samples and stored in a sealed container at room temperature until further use. Compositional analyses of the cladode flour, which included total solids, ash, cellulose, structural carbohydrates, lignin and extractives, was determined using standard biomass analytical procedures described by the National Renewable Energy Laboratory (2012). The starch content was determined through starch hydrolysis as per the protocol of the manufacturer of the Megazyme K-TSTA assay kit (Megazyme, Bray, Ireland). The nitrogen content was determined by a standard micro-Kjeldahl procedure and multiplied by 6.25 to calculate the crude protein content (AOAC 2005).

### Biomass pretreatment

Dilute acid pretreatment of the *O*. *ficus*-*indica* cladode biomass was performed by first mixing 2.5 kg of cladode flour with 8.4 l of a 1.5 % (w/w) solution of H_2_SO_4_ to achieve a solids loading of 30 % (w/v) in a 15 l stainless steel Biostat-C bioreactor (Sartorius Stedim Biotech, Göttingen, Germany). The slurry was left overnight and subsequently heated in situ to 120 °C for 50 min prior to use for SHF experiments. A similar but slightly scaled down approach in a Biostat B-plus bioreactor with a 1.6 l glass vessel (Sartorius Stedim Biotech) was used to obtain the pretreated slurry used for SSF experiments, but using a Hiclave HV-110 autoclave (Hirayama, Saitama, Japan) for autoclaving the vessel containing the slurry. The pretreatment conditions had been previously optimized by means of a central composite design using dilute acid concentration and pretreatment time as variables, at a fixed temperature of 120 °C (Kuloyo 2012).

### Enzymes and enzyme assays

Celluclast 1.5L (cellulase), Novozyme 188 (β-glucosidase) and Pectinex Ultra SP-L (pectinase) preparations were kindly donated by Novozymes A/S (Bagsvaerd, Denmark). The cellulase and β-glucosidase activities of the above enzyme preparations were determined using standard assays (Ghose 1987); cellulase activity was expressed as filter paper units, FPU ml^−1^, whereas the β-glucosidase activity was expressed as international units (IU), where 1 IU is equal to 1 μmol min^−1^ of substrate converted. Pectinase activity was determined as described elsewhere (Phutela et al. 2005), using citrus pectin (Sigma-Aldrich, St. Louis, MO, USA) as substrate and galacturonic acid (Sigma-Aldrich) as standard and expressed as units (U) where 1 U is equal to the amount of enzyme required to release 1 μmol min^−1^ of galacturonic acid equivalents.

### Production of cladode enzymatic hydrolysate for SHF

To produce an *O*. *ficus*-*indica* cladode enzymatic hydrolysate for SHF, the whole pretreated slurry was used without separating the liquid fraction from the water insoluble solids. Following dilute acid pretreatment, the temperature of the slurry was decreased to 50 °C and the pH adjusted to and maintained at pH 5. Enzymatic hydrolysis was performed by adding an enzyme cocktail containing 15 FPU of cellulase, 15 IU of β-glucosidase and 100 U of pectinase per g dry biomass to the pretreated slurry, which was stirred at 300 rev min^−1^ for 48 h to allow maximum release of monomeric sugars. Subsequently, the resulting hydrolysate slurry was collected in sterile 1 l bottles that were stored at −20 °C until further use.

### Yeast strains and inoculum preparation

*K*. *marxianus* UOFS Y-2791, isolated from an agave plant (*Agave americana*), and *S*. *cerevisiae* UOFS Y-0528, a commercial wine yeast strain, were obtained from the University of the Free State MIRCEN yeast culture collection. Axenic cultures of these strains were maintained at 4 °C on GPY agar slants, containing (per litre) 40 g glucose, 5 g peptone, 5 g yeast extract and 20 g agar. Pre-cultures of *K*. *marxianus* and *S*. *cerevisiae* were prepared in a sterile medium containing (per litre) 30 g glucose, 0.25 g citric acid, 3 g yeast extract, 5 g (NH_4_)_2_SO_4_, 9.6 g KH_2_PO_4_, 0.76 g K_2_HPO_4_, 0.75 g MgSO_4_·7H_2_O, 0.05 g CaCl_2_·2H_2_O, 0.1 g NaCl and 1 ml of a trace elements stock solution that contained (per litre) 0.035 g FeSO_4_·7H_2_O, 0.007 g MnSO_4_·7H_2_O, 0.011 g ZnSO_4_·7H_2_O, 0.001 g CuSO_4_·5H_2_O, 0.002 g CoSO_4_·6H_2_O, 0.0,013 g Na_2_MoO_4_·2H_2_O, 0.002 g H_3_BO_3_, 0.0004 g KI and 0.0016 g Al_2_(SO_4_)_3_·18H_2_O. The pH of the medium was adjusted to pH 5.5 by the addition of 3 M KOH. A loopful of cells from 24 h agar slants was inoculated into 500 ml side-arm flasks with cotton wool plugs containing 50 ml of the above growth medium and incubated at 34 °C on an orbital shaker at 200 rev min^−1^. These *K*. *marxianus* and *S*. *cerevisiae* pre-cultures were incubated for 9 and 11 h, respectively, until the cells had reached the late exponential growth phase. Active cultures for inoculating the fermentation medium were subsequently prepared by inoculating 1 ml from each pre-culture into another set of shake flasks containing the same medium. These were also incubated until late exponential phase and immediately used to inoculate a chemically defined fermentation medium, the enzymatic hydrolysate for SHF experiments or the pretreated slurry for SSF experiments.

### Chemically defined fermentation medium

A chemically defined medium containing a sugar mixture similar in composition to the enzymatic hydrolysate of the *O*. *ficus*-*indica* cladodes was initially used to evaluate the performance of both yeast strains as a benchmark before proceeding with the fermentation of the actual cladode hydrolysate. This medium contained (per litre) 0.5 g citric acid, 5 g (NH_4_)_2_HPO_4_, 0.75 g KH_2_PO_4_, 0.5 g K_2_HPO_4_, 0.2 g MgSO_4_·7H_2_O, 0.02 g CaCl_2_·2H_2_O, 0.1 g NaCl, 1 ml of a trace elements stock solution as described above and 4 ml of a vitamin stock solution. The latter contained (per litre) 0.025 g biotin, 0.5 g calcium pantothenate, 0.5 g nicotinic acid, 0.1 g *p*-aminobenzoic acid, 0.5 g pyridoxine hydrochloride, 0.5 g thiamine hydrochloride, and 12.5 g *m*-inositol (all vitamins from Sigma-Aldrich). The sugar mixture was autoclaved separately, whereas the mineral salts solution was autoclaved in the bioreactor at 121 °C for 20 min after adjustment to pH 5.0 by titration with 3 M KOH. The filter-sterilized vitamin stock solution was added to the sterile sugar mixture and aseptically transferred into the bioreactor.

### Fermentation conditions

Fermentations of the chemically defined medium and the hydrolysate were performed in 1.6-l Biostat B-plus stirred tank reactors (Sartorius Stedim Biotech), each fitted with an exhaust gas condenser operated at 4 °C and using a 1 l culture volume. The nutrient supplementation of the enzymatic hydrolysate used for SHF and the acid-pretreated slurry used for SSF were similar to those used for the chemically defined medium. The inoculum size of 5 % (v/v) gave an initial optical density at 690 nm of 0.26, equivalent to a *K*. *marxianus* dry biomass concentration of 0.12 and 0.1 g l^−1^ in the case of *S*. *cerevisiae*. The culture was maintained at pH 5.0 by automatic titration with either 3 M KOH or 1.5 M H_2_SO_4_. *K*. *marxianus* was grown at 40 °C and *S*. *cerevisiae* at 35 °C. These temperatures were based on the upper limit of the optimum growth temperature range determined from temperature profile experiments of these two yeast strains (unpublished results). The fermentation temperature used for *K*. *marxianus* was close to the optimum temperature of between 45 and 50 °C for enzymatic hydrolysis of the constituent carbohydrate polymers of the cladode biomass. Both yeasts were cultivated under non-aerated conditions and *K*. *marxianus* under oxygen-limited conditions as well. During non-aerated cultivation the culture was kept homogenous by maintaining a constant slow stirrer speed of 100 rev min^−1^, whereas during oxygen-limited cultivation the culture was sparged with sterile air at a low aeration rate of 0.3 l min^−1^ with the dissolved oxygen tension (DOT) maintained at 0.5–1 % of saturation by automatic adjustment of the stirrer speed. All fermentations were carried out in at least duplicate and mean values are reported.

### SHF and SSF procedures

An 800 ml volume of the enzymatic hydrolysate produced for SHF was sterilized by mild autoclaving at 110 °C for 10 min, cooled to the desired cultivation temperature and supplemented with 150 ml of a sterile nutrient and vitamin solution to give concentrations similar to the chemically defined medium. The nutrient solution also served as diluent to obtain a more miscible slurry. A 5 % (v/v) inoculum was transferred to the reactor vessel, giving a 1 l working volume and an initial biomass concentration of 0.16 or 0.15 g l^−1^ in the case of *K*. *marxianus* and *S*. *cerevisia*e, respectively.

SSF experiments were performed in a similar fashion as the SHF experiments, but using the whole slurry directly from the pretreatment stage. After cooling to 40 °C and adjustment to pH 5.0, the slurry was supplemented with the above nutrients and sterile deionized water added to ensure a final water insoluble solids concentration of 140 g l^−1^, thereby conforming to the same solids loading used during the hydrolysis stage of SHF. A time interval of about 10 min between the addition of the enzyme mixture and inoculation was allowed for the viscosity to decrease to facilitate better mixing. The stirrer speed was decreased from 300 to 150 rev min^−1^ prior to inoculation. To enable comparison of the ethanol yields obtained in SSF and SHF experiments, the final sugar concentrations obtained following enzymatic hydrolysis during SHF experiments were used to calculate the ethanol yields on total sugars.

### Analytical procedures

The composition of the exhaust gas during fermentation was continuously monitored using an Uras 10E infrared CO_2_ and a Magnos 6G paramagnetic O_2_ analyser (Hartman and Braun, Frankfurt, Germany). Cell concentrations during fermentation in chemically-defined medium were monitored by measuring culture turbidity against a medium blank at 690 nm with a Photolab S6 photometer (WTW, Weilheim, Germany). Dry cell weight was gravimetrically determined using duplicate 10 ml samples that were centrifuged, washed with distilled water and dried overnight at 105 °C. Ethanol concentration was determined with a Shimadzu GC 2010 gas chromatograph (Shimadzu Scientific Instruments, Columbia, MD, USA) equipped with a Phenomenex ZB wax column (Phenomenex, Torrance, CA, USA) and a flame ionization detector, with hydrogen as carrier gas at 35 cm^3^ min^−1^. The oven temperature was 80 °C for 2.5 min, ramped at 25 °C min^−1^ to 180 °C with a 2 min isothermal period. The injection volume was 0.6 µl at a 50:1 split ratio. The inlet and detector temperatures were 150 and 300 °C, respectively. Samples collected for determining sugar utilization and product formation were immediately cooled in ice before centrifugation at 10,600×*g* at 4 °C. Prior to chromatographic analyses, samples of the slurry resulting from the pretreatment procedure were neutralized with Ca(OH)_2_ to precipitate the sulphates, whereas enzymatic hydrolysate slurries were diluted to reduce the viscosity of the sample and prolong the life of the chromatographic column. All supernatants were filtered through a 0.45 µm membrane filter prior to chromatographic analysis. Supernatants not immediately analysed were stored at −20 °C. The concentrations of glucose, xylose galactose, arabinose and fructose were determined by HPLC with automatic injection of 10 µl samples, using a Rezex RPM-monosaccharide Pb+2 cation exchange column (Phenomenex) equipped with a guard column and operated at an oven temperature of 85 °C using deionized water as the mobile phase at a flow rate of 0.4 ml min^−1^. Individual and mixed pure sugar solutions were used as standards for the quantification of sugars.

## Results

### Composition of *O*. *ficus*-*indica* cladode biomass

The chemical composition of *O*. *ficus*-*indica* cladode powder, on a dry weight basis, is shown in Table 1. The total sugar content of 42 %, especially the glucan content, was less than that of some other conventional lignocellulosic feedstocks. However, this study showed that the *O*. *ficus*-*indica* cladode biomass had a high content of galactan and fructan that compensated to some extent for the low glucan content. Their monomeric units (galactose and fructose) are readily fermentable and, together with glucose, these sugars accounted for 34.3 % of the dry cladode biomass, which was comparable to the content of fermentable sugars of other feedstocks such as sugar cane bagasse (42.1 %) (Neureiter et al. 2002), corn stover (39 %) (National Renewable Energy Laboratory 2013) and barley straw (38.3 %) (García-Aparicio et al. 2011) (Table 1). The low xylan content of the cladode biomass agreed with the findings reported by Ginestra et al. (2009). The cladode lignin content of 7.95 % was substantially lower than that of other feedstocks and this could result in the formation of less toxic inhibitors such as phenolic compounds as a consequence of dilute acid pretreatment. The biomass had a high content of extractives (24.3 % dry wt), which HPLC analysis indicated consisted of mainly galacturonic acid subunits. The composition of other constituents such as ash, lignin, and protein (Table 1) were similar to previous analytical data on the *O*. *ficus*-*indica* cladode (Stintzing and Carle 2005).

**Table 1:** Mean chemical composition (± SD of the mean) of *O*. *ficus*-*indica* cladodes in weight per cent of dry biomass, compared to some conventional lignocellulosic feedstocks

Constituent (% dry wt)	*O*. *ficus*-*indica* cladode (this study)	*O*. *ficus*-*indica* cladode^a^	Sugar cane bagasse^b^	Corn stover^c^	Barley straw^d^
*Glucan	23.1 ± 1.3	15.3	40.2	37.7	37.1
Xylan	3.9 ± 0.4	1.9	22.5	21.6	21.3
Arabinan	3.8 ± 0.3	4.0	2.0	2.4	3.8
*Galactan	6.4 ± 0.6	3.4	1.4	0.9	1.2
*Fructan	4.8 ± 0.4	–	–	–	–
Fucan	–	0.07			
*Mannan	Trace	1.4	0.5	0.4	–
Rhamnan	–	0.7	–	–	–
Total sugars	42.0	26	66.6	63.0	63.4
Total fermentable sugars	34.3	19.7	42.1	39.0	38.3
Lignin	7.9 ± 0.8	16	25.2	18.6	19.2
Ash	16.8 ± 0.2	n/a	10–15	10.1	8.2
Protein	7.5 ± 0.2	6.42	n/a	n/a	n/a
Extractives	24.3 ± 1.1	17.7	n/a	5.6	15.4
Total	98.5				

* Readily fermentable constituent sugars

^a^(Ginestra et al. 2009) The author used a mixture of three cultivars, Surfarina, Muscaredda and Sanguigna, which were cultivated in Italy

^b^(National Renewable Energy Laboratory 2013)

^c^(National Renewable Energy Laboratory 2013)

^d^(García-Aparicio et al. 2011)

### Pretreatment and enzymatic hydrolysis

Dilute acid pretreatment of the biomass yielded a hydrolysate slurry containing (per litre) 7.4 g glucose, 3.9 g xylose, 2.3 g galactose, 4.0 g arabinose and 5.0 g fructose. A high solids loading of 30 % (w/v) was used to produce a hydrolysate with sugar content close to the values reported for hydrolysates of other conventional lignocellulosic biomass feedstocks. Enzymatic hydrolysis with cellulase, β-glucosidase and pectinase was performed directly on the whole pretreated cladode slurry, which released 78 % of the theoretical sugars and close to 80 % of the theoretical glucose in the biomass. The hydrolysate used for subsequent SHF experiments contained (per litre) 45.5 g glucose, 6.3 g xylose, 9.1 g galactose, 10.8 g arabinose and 9.6 g fructose. Thus, the total monosaccharide concentration was 81.3 g l^−1^, of which 64.2 g l^−1^ were readily fermentable sugars (glucose, galactose and fructose).

### Fermentation of a simulated cladode hydrolysate

The fermentation of a simulated cladode hydrolysate served as benchmark for the performance of *K*. *marxianus* and *S*. *cerevisiae* in the actual hydrolysate. In the non-aerated cultures of *K*. *marxianus* the DOT dropped from 100 to 0.2 % of saturation within 30 min after inoculation due to cell respiration. No precautions, such as sparging with nitrogen gas, were taken to ensure strict anaerobic conditions. Therefore, because of oxygen diffusion into the culture vessel through the silicone rubber tubing, aided by continuous stirring of the culture broth at 100 rev min^−1^, the rate of oxygen diffusion into the culture was sufficient for ethanol production and some growth, albeit at very low rates. The respiro-fermentative metabolism exhibited by *K*. *marxianus* has also been reported with the related species *K*. *lactis*, when grown under similar conditions (Merico et al. 2009).

**Fig. 1:**
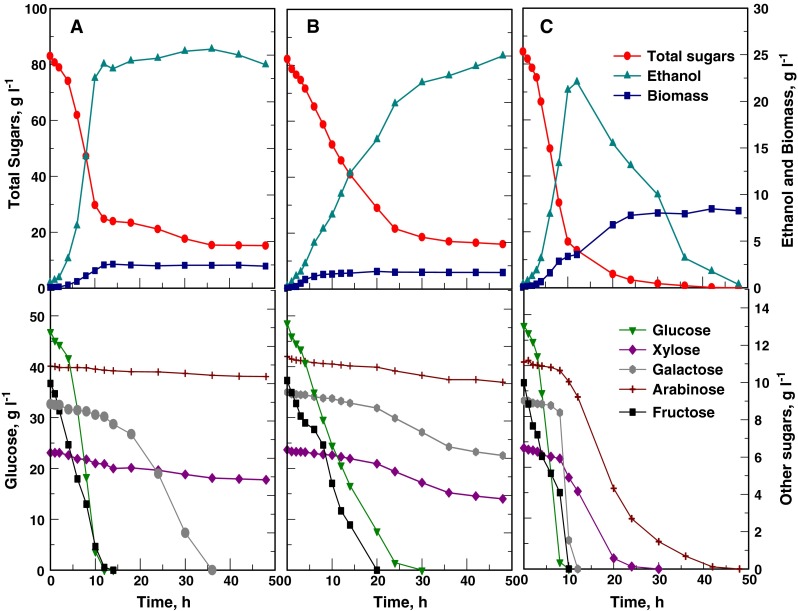
Fermentation profiles of *S*. *cerevisiae* Y-0528 and *K*. *marxianus* Y-2791 in a chemically defined medium containing a sugar mixture resembling an enzymatic hydrolysate of *O*. *ficus*-*indica* cladode biomass using different aeration conditions. **a** *S*. *cerevisiae,* non-aerated. **b** *K*. *marxianus,* non-aerated, and **c** *K*. *marxianus,* oxygen-limited cultivation

**Fig. 2:**
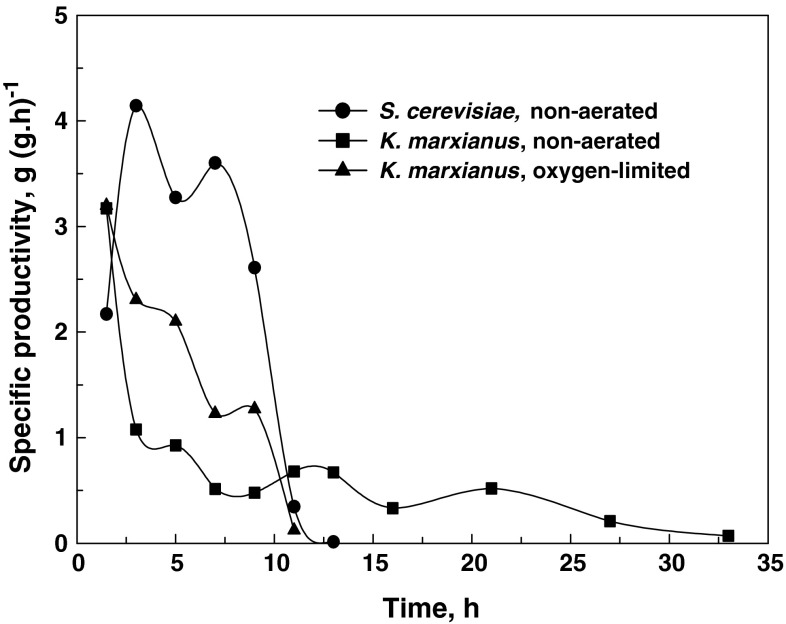
Profiles of specific ethanol productivity versus time during cultivation of *S*. *cerevisiae* and *K*. *marxianus* under non-aerated and oxygen-limited conditions in a chemically defined medium containing a sugar mixture simulating an enzymatic hydrolysate of *O*. *ficus*-*indica* cladode biomass

Non-aerated cultivation of *S*. *cerevisiae* resulted in a maximum ethanol concentration of 25.8 g l^−1^ (mean value) after 36 h at 35 °C, whereas 25.0 g l^−1^ was obtained with *K*. *marxianus* at 40 °C after 48 h. The maximum volumetric ethanol productivity (*Q*_*p*_) of *K*. *marxianus* was almost four-fold lower than that of *S*. *cerevisiae* (Fig. 1; Table 2), in addition to a much lower ethanol specific productivity (*q*_*p*_) in the range of 0.33 and 0.93 g g^−1^ h^−1^ between 5 and 21 h of fermentation (Fig. 2). *S*. *cerevisiae* had *q*_*p*_ values that ranged between 3.27 and 4.14 g g^−1^ h^−1^ during the first 7 h of fermentation. Ethanol yield coefficients of 0.38 and 0.39 on sugars utilized were obtained with *K*. *marxianus* and *S*. *cerevisiae*, respectively. The ethanol yield coefficients on total sugars were also comparable (Table 2). *K*. *marxianus* produced substantially less biomass than S. *cerevisiae* (Fig. 1a, b; Table 2). Over a brief initial time period *K*. *marxianus* exhibited a maximum specific growth rate (µ_max_) of 0.47 h^−1^, giving a maximum volumetric biomass productivity (*Q*_*p biomass*_) of 0.39 g l^−1^ h^−1^, compared to the corresponding values of 0.38 h^−1^ and 0.27 g l^−1^ h^−1^ obtained with *S*. *cerevisiae* (Table 2).

**Table 2:** Fermentation parameters of *S*. *cerevisiae* Y-0528 and *K*. marxianus Y-2791 in a chemically defined medium containing a sugar mixture resembling an enzymatic hydrolysate of *O*. *ficus*-*indica* cladode biomass

Parameter	*S*. *cerevisiae*	*K*. *marxianus*	
	Non-aerated	Non-aerated	Oxygen-limited
Residual sugars, g l^−1^	15.3	18.2	0.0
µ_max_, h^−1^	0.38	0.47	0.52
Q_p_, g ethanol l^−1^ h^−1^	3.28	0.85	2.98
Q_p_, g biomass l^−1^ h^−1^	0.27	0.39	0.55
Maximum ethanol, g l^−1^	25.8	25.0	22.1
Maximum dry biomass, g l^−1^	2.6	1.90	8.5
Ethanol yield on utilized sugars	0.39	0.38	0.31
Ethanol yield on total sugars	0.31	0.30	0.26
Biomass yield on utilized sugars	0.04	0.04	0.1
Biomass yield on total sugars	0.03	0.03	0.1

Mean values (which were within ±2 % of the individual values) of duplicate experiments are shown

When *K*. *marxianus* was cultivated under oxygen-limited conditions, obtained by using a low rate of air sparging and controlling the DOT at 0.5–1 % of saturation, an ethanol concentration of 22.1 g l^−1^ was reached after 12 h of cultivation. The *Qp* values of *K*. *marxianus* were comparable to those of *S*. *cerevisiae* (Table 2) due to an increased specific ethanol productivity (Fig. 2) and biomass concentration (Fig. 1c). The drawback of growing *K*. *marxianus* with aeration was that the ethanol produced was utilized for *biomass* production as soon as the hexoses were depleted (Fig. 1c), despite the low DOT.

Under the above two aeration conditions, both yeasts utilized glucose and fructose preferentially, as expected, whereas galactose uptake was minimal prior to depletion of these sugars (Fig. 1). In contrast with the non-aerated fermentation profile, under oxygen-limited conditions *K*. *marxianus* completely utilized galactose as well as xylose and arabinose. The rate of pentose utilization increased after depletion of the hexoses (Fig. 1c).

### SHF of an enzymatic hydrolysate of *O*. *ficus*-*indica* cladode biomass

**Fig. 3:**
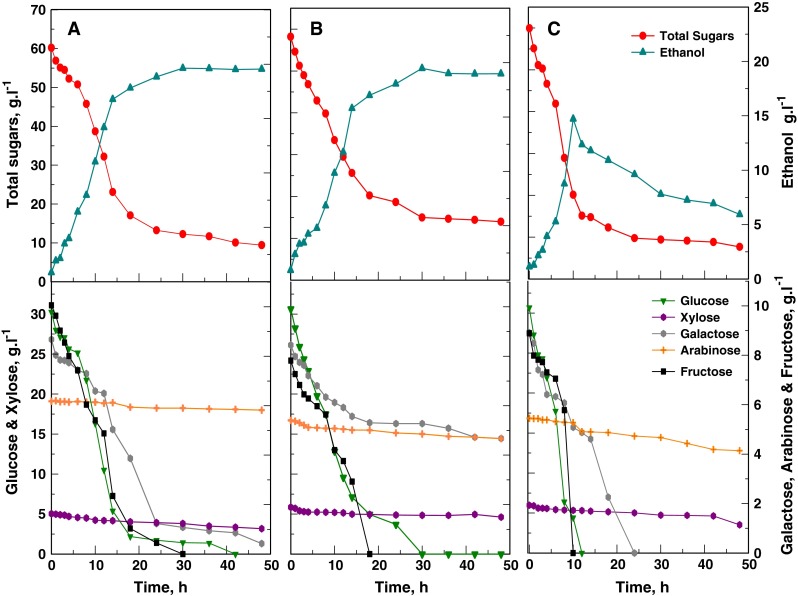
SHF profiles of *S*. *cerevisiae* Y-0528 and *K*. *marxianus* Y-2791 in *O*. *ficus*-*indica* cladode hydrolysate using different aeration conditions. **a** *S*. *cerevisiae,* non-aerated. **b** *K*. *marxianus,* non-aerated. **c** *K*. *marxianus,* oxygen-limited. Time zero indicates the time of inoculation following 48 h of enzymatic hydrolysis at 50 °C

SHF fermentation profiles of *K*. *marxianus* and *S*. *cerevisiae* under non-aerated conditions, as well as that of *K*. *marxianus* under oxygen-limited conditions, are shown in Fig. 3. The hydrolysate medium used in each SHF experiment had a total sugar content of 59.5 g l^−1^, consisting of (per litre), 30.7 g glucose, 5.6 g xylose, 8.7 g galactose, 5.9 g arabinose and 8.6 g fructose. This sugar concentration was lower than in the chemically-defined medium as a result of the hydrolysate having been slightly diluted to obtain a more miscible slurry. *S*. *cerevisiae* and *K*. *marxianus*, grown in the absence of aeration, produced similar maximum ethanol concentrations of 19.6 and 19.5 g l^−1^ (mean values), respectively, corresponding to ethanol yields of about 80 and 66 % of the theoretical yield based on utilized sugars and total sugars, respectively (Fig. 3; Table 3). However, under these conditions *K*. *marxianus* gave a maximum volumetric productivity of 0.93 g ethanol l^−1^ h^−1^, which was lower than that of *S*. *cerevisiae* (1.48 g l^−1^ h^−1^). The sugar utilization pattern in these SHF experiments was similar to the trends found in the chemically defined medium (Figs. 1, 3).

**Table 3:** Fermentation parameters of *S*. *cerevisiae* and *K*. *marxianus* during SHF and SSF of an *O*. *ficus*-*indica* cladode hydrolysate. Mean values (which were within ±2 % of the individual values) of duplicate experiments are shown

Parameter	SHF			SSF		
	*S*. *cerevisiae*	*K*. *marxianus*		*S*. *cerevisiae*	*K*. *marxianus*	
	Non-aerated	Non-aerated	Oxygen-limited	Non-aerated	Non-aerated	Oxygen-limited
Residual sugars, g l^−1^	9.4	13.5	7.7	5.4	13.3	2.7
Q_p (EtOH)_, g l^−1^ h^−1^	1.48	0.93	2.23	1.41	1.08	1.57
Q_p__(overall)_, g l^−1^ h^−1^	0.25	0.25	0.24	0.57	0.54	0.71
Max. ethanol, g l^−1^	19.6	19.5	14.7	20.6	19.3	14.2
Y_EtOH_, utilized sugars	0.40	0.42	0.28	nd	nd	nd
Y_EtOH_, total sugars	0.33	0.33	0.25	0.35	0.3	0.24

*nd* not determined

Under oxygen-limited conditions *K*. *marxianus* completely consumed galactose (Fig. 3c). Even under these conditions xylose and arabinose were poorly utilized, although the uptake of xylose and, to a lesser extent, arabinose, proceeded faster towards the end of the fermentation. This was in contrast to cultivation in a chemically-defined medium where both sugars were completely consumed (Fig. 1c). *K*. *marxianus* produced an ethanol concentration of 14.7 g l^−1^ compared to the 19.5 g l^−1^ obtained under non-aerated conditions (Fig. 3). Ethanol assimilation was evident after depletion of the glucose and fructose. Nevertheless, *K*. *marxianus* achieved a maximum volumetric ethanol productivity of 2.23 g ethanol l^−1^ h^−1^, which was more than double the value obtained during non-aerated cultivation and which also exceeded that of *S*. *cerevisiae* (Table 3).

### SSF of pretreated *O*. *ficus*-*indica* cladode biomass

**Fig. 4:**
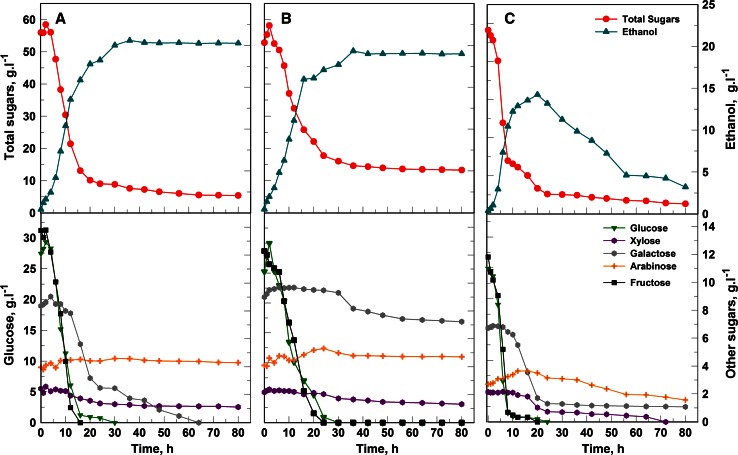
SSF profiles of *S*. *cerevisiae* Y-0528 and *K*. *marxianus* Y-2791 in *O*. *ficus*-*indica* cladode hydrolysate using different conditions of aeration. **a** *S*. *cerevisiae,* non-aerated, **b** *K*. *marxianus,* non-aerated, **c** *K*. *marxianus,* oxygen-limited. Time zero indicates the time of inoculation, which was 10 min after the addition of enzymes

Substantial saccharification already occurred during the 10 minute interval between enzyme addition and yeast inoculation for SSF, resulting in about 55 g l^−1^ of monomeric sugars being released, including about 25 g glucose l^−1^ (Fig. 4). A similar rapid release of monomeric sugars was observed during the enzymatic hydrolysis step of SHF (results not shown). The total sugar concentration in the hydrolysate continued to increase during the first few hours of fermentation, mainly due to the accumulation of glucose, indicating that glucan hydrolysis was occurring faster than glucose uptake for growth and ethanol production (Fig. 4). The viscosity of the hydrolysate gradually decreased over time, presumably because of the loss of water binding capacity due to cellulose degradation (Rosgaard et al. 2007) as well as pectin solubilization. After 36 h of non-aerated cultivation, *S*. *cerevisiae* produced an ethanol concentration of 20.6 g l^−1^, whereas *K*. *marxianus* achieved a slightly lower concentration of 19.3 g l^−1^. These values were similar to those obtained during SHF and corresponded to 70 and 64 %, respectively, of the theoretical yield on total sugars in the hydrolysate.

Significant galactose uptake commenced only after glucose and fructose were almost completely depleted (Fig. 4). As found in SHF experiments and with the chemically defined medium, *K*. *marxianus* poorly utilized galactose, xylose and arabinose under non-aerated conditions, whereas they were better utilized under oxygen-limited conditions. Ethanol production with *K*. *marxianus* under oxygen-limited conditions peaked at 20 h, reaching 14.2 g l^−1^ (Fig. 4c). Again, the ethanol produced was assimilated following glucose and fructose depletion. Under these non-aerated conditions, *K*. *marxianus* exhibited a maximum volumetric ethanol productivity of 1.08 g l^−1^ h^−1^, whereas *S*. *cerevisiae* performed considerably better, achieving 1.41 g l^−1^ h^−1^ (Fig. 4a, b; Table 3). Of the two aeration conditions, the highest maximum volumetric ethanol productivity of 1.57 g l^−1^ h^−1^ was achieved with *K*. *marxianus* under oxygen-limited conditions, although this value was almost 1.5-fold lower than obtained during SHF under similar aeration conditions. However, under both non-aerated and oxygen-limited conditions, the overall ethanol productivity achieved with either yeast using SSF was more than double the values recorded for SHF (Table 3).

## Discussion

The high water content of 88–95 % (w/w) of fresh *O*. *ficus*-*indica* cladodes was the first challenge in this study. This problem was addressed by first sun-drying and milling the cladode, giving a more concentrated raw material with a moisture content of 3.7 ± 0.1 %, which also facilitated storage and handling. The cladode biomass was rich in pectin, which is hydrolysed into galacturonic acid units by pectinase. Although no enzymatic hydrolysis of the pretreated slurry was performed using pectinase alone, it is believed that the inclusion of pectinase in the enzyme cocktail facilitated a decreased viscosity of the hydrolysate. A mixture of pectinase and cellulase enzymes was necessary for pectin solubilization and the complete release of sugars in orange peel (Grohmann et al. 1995). Nevertheless, separation of the solid and liquid fractions of the slurry resulting from the acid pretreatment of the cladode biomass proved difficult due to the small size of the biomass particles and it was too viscous to filter without further dilution. Therefore, enzymatic hydrolysis was performed directly on the whole pretreated cladode slurry.

The low xylose and negligible mannose content of the cladode biomass used in this study suggested that the hemicellulose structure of the *O*. *ficus*-*indica* cladode was atypical of hardwood or softwood hemicelluloses. Because ethanol production from the cladode biomass would, therefore, be mainly based on its hexose content, a xylose fermenting yeast strain may not necessarily be a requisite for an economically viable bioprocess.

The observations that ethanol production commenced immediately after inoculation and that the ethanol yield coefficients were similar to those obtained in the chemically defined medium, suggested that the yeasts had adapted well to the conditions in the hydrolysate and that the levels of inhibitory compounds such as furfural and hydroxymethylfurfural, arising from sugar degradation during acid pretreatment, were minimal. Zhang et al. (2010) reported that inhibitors in corncob and soybean cake hydrolysates resulted in low ethanol yields by *K*. *marxianus* CBS 6556. *K*. *marxianus* has been shown to have a lower tolerance to ethanol than *S*. *cerevisiae* (Banat and Marchant 1995; Rosa and Sa-Correia 1992). The ethanol tolerance of *S*. *cerevisiae* Y-0528 in terms of growth was 112 g l^−1^, this value representing the upper limit for growth, whereas the ethanol tolerance of *K*. *marxianus* Y-2791 was determined as 84 g l^−1^ (unpublished data). Nevertheless, the latter value exceeded the ethanol concentrations likely to be reached during the fermentation of lignocellulosic hydrolysates. Therefore, the ethanol tolerance of *K*. *marxianus* Y-2791 was deemed sufficient for use of this yeast in the fermentation of lignocellulosic biomass feedstocks.

With both *S*. *cerevisiae* and *K*. *marxianus*, SSF proved vastly superior to SHF in respect of the rate of ethanol production, with comparable ethanol yield coefficients. A similar finding was reported by Zhang et al. (2010) in one of the few investigations using *K*. *marxianus* for the fermentation of lignocellulosic feedstocks, who reported that SSF of corncob and soybean cake hydrolysates using *K*. *marxianus* CBS 6556 resulted in the highest ethanol production of 5.68 and 2.14 g l^−1^, respectively. Unfortunately the latter paper contained insufficient data for a detailed comparison of fermentation parameters. In contrast to the low efficiency of ethanol production by *K*. *marxianus* reported by these authors, our experiments showed that under oxygen-limited conditions, achieved by controlling the DOT at between 0.5 and 1 % of saturation, *K*. *marxianus* was capable of a volumetric ethanol productivity and yield comparable to *S*. *cerevisiae*. However, even this low dissolved oxygen level was sufficient for *K*. *marxianus* to utilize the produced ethanol as a carbon source following glucose depletion. The disappearance of about 1.5 g xylose l^−1^ from the medium during the cultivation of *S*. *cerevisiae* suggested that this strain had some capacity for xylose uptake. In fact, it has previously been reported that *S*. *cerevisiae* utilized xylose in conjunction with ribose, glucose, glycerol or galactose (Van Zyl et al. 1989). Further investigation is required to confirm if this was also valid for our strain. The apparent decrease of 0.6 g l^−1^ in the arabinose concentration might have been due to HPLC analytical error, rather than uptake by *S*. *cerevisiae*.

The poor uptake of galactose by *K*. *marxianus* under non-aerated conditions may have been due to the yeast exhibiting the Kluyver effect which, simply put, is the inability of yeasts to effectively ferment certain sugars in the absence of oxygen or respiration (Fukuhara 2003). The rationale behind this respiration-dependent assimilation of certain sugars is still not clear, but it is believed to be due to the interplay of several factors involving a decreased rate of transport and metabolism of certain sugars under anaerobic conditions (Fukuhara 2003; Goffrini et al. 2002; Rodrussamee et al. 2011). *S*. *cerevisiae* does not exhibit a Kluyver effect on galactose. It has been suggested that the poor utilisation of the pentoses xylose and arabinose by *K*. *marxianus* in the absence of aeration was due to the lack of NADH oxidation by the mitochondrial electron transport chain (Rodrussamee et al. 2011). Neither of the two yeasts used in this study grew on galacturonic acid as the sole carbon source (results not shown).

In conclusion, the ethanol concentration of 2.6 % (w/v) we obtained by fermentation of the *O*. *ficus*-*indica* cladode hydrolysate was an improvement on the 1.4 % (w/v) previously reported in the only other published paper, to our knowledge, on ethanol production from *O*. *ficus*-*indica* cladodes (Retamal et al. 1987). Limited aeration enhanced the volumetric ethanol productivity of *K*. *marxianus* and the utilization of galactose, xylose & arabinose. It was demonstrated that *K*. *marxianus* had potential as an alternative to *S*. *cerevisiae* for bioethanol production, although using an optimal aeration regime was essential to maximize its rate and yield of ethanol production. However, further bioprocess development, especially to deal with the high viscosity of the cladode biomass slurry and to increase the fermentable carbohydrate concentration in the hydrolysate, is required to obtain an economically viable ethanol concentration of at least 4 % (w/v) (Wingren et al. 2003) from this lignocellulosic feedstock.
